# Bluetongue Virus Capsid Assembly and Maturation

**DOI:** 10.3390/v6083250

**Published:** 2014-08-21

**Authors:** Bjorn-Patrick Mohl, Polly Roy

**Affiliations:** Department of Pathogen Molecular Biology, Faculty of Infectious and Tropical Diseases, London School of Hygiene and Tropical Medicine, Keppel Street, London, WC1E 7HT, UK; E-Mail: Bjorn.Mohl@lshtm.ac.uk

**Keywords:** Capsid assembly, in vitro assembly, recombinant protein, Cryo-EM

## Abstract

Maturation is an intrinsic phase of the viral life cycle and is often intertwined with egress. In this review we focus on orbivirus maturation by using Bluetongue virus (BTV) as a representative. BTV, a member of the genus Orbivirus within the family Reoviridae, has over the last three decades been subjected to intense molecular study and is thus one of the best understood viruses. BTV is a non-enveloped virus comprised of two concentric protein shells that encapsidate 10 double-stranded RNA genome segments. Upon cell entry, the outer capsid is shed, releasing the core which does not disassemble into the cytoplasm. The polymerase complex within the core then synthesizes transcripts from each genome segment and extrudes these into the cytoplasm where they act as templates for protein synthesis. Newly synthesized ssRNA then associates with the replicase complex prior to encapsidation by inner and outer protein layers of core within virus-triggered inclusion bodies. Maturation of core occurs outside these inclusion bodies (IBs) via the addition of the outer capsid proteins, which appears to be coupled to a non-lytic, exocytic pathway during early infection. Similar to the enveloped viruses, BTV hijacks the exocytosis and endosomal sorting complex required for trafficking (ESCRT) pathway via a non-structural glycoprotein. This exquisitely detailed understanding is assembled from a broad array of assays, spanning numerous and diverse *in vitro* and *in vivo* studies. Presented here are the detailed insights of BTV maturation and egress.

## 1. Introduction

Members of the family *Reoviridae*, which include BTV and other orbiviruses, are characterized by their unique genome of 10–12 segments of linear, double-stranded RNA (dsRNA). These separate segments facilitate the generation of 10–13 viral proteins. Virions are comprised of non-enveloped, icosahedral capsids. The structural organization of the virions entails the enclosure of RNA gene segments within two concentric protein shells, which gives rise to a distinct outer capsid layer that surrounds an inner capsid or core that contains the genome. Following cell entry, the outer capsid dissociates, this facilitates the release of the core within which the viral genome remains sequestered into the cytoplasm. The retention of the genome within this core abrogates the triggering of an innate immune response. Given this sequestration of the viral genome, the cores contain the necessary transcription machinery, which facilitates the synthesis and subsequent extrusion of multiple capped positive-sense RNAs, derived from each genomic segment, into the cytoplasm. Current models hold that the polymerase complex contained within this core contacts the dsRNA genome, which, acting as a template, facilitates the synthesis of nascent transcripts that are directed out of the core particle via pores. Such mechanizations require the co-ordination of enzymatic activities that include polymerase, helicase and RNA capping activity. Whilst intense efforts on investigating viral architecture and structure have yielded a trove of information, the underlying dynamics of subsequent core maturation and egress remain to be fully elucidated and defined; the use of BTV provides an opportunity to do so.

**Figure 1:**
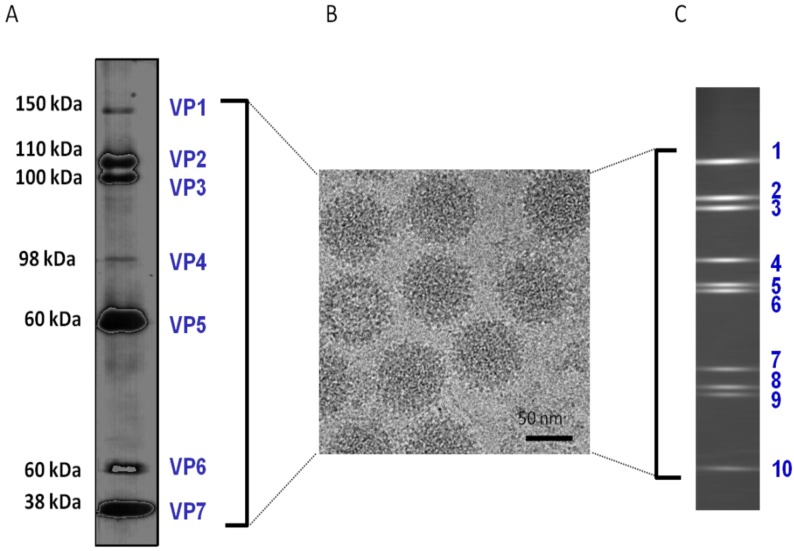
BTV (Reoviridae) particle constituents. (**A**) SDS-PAGE showing the 7 structural proteins isolated from purified virions, in addition, 4 non-structural proteins are synthesized in infected cells. (**B**) Transmission electron micrograph (TEM) of BTV virions. (**C**) PAGE showing 10 discrete double-stranded RNA molecules derived from purified virions.

**Figure 2:**
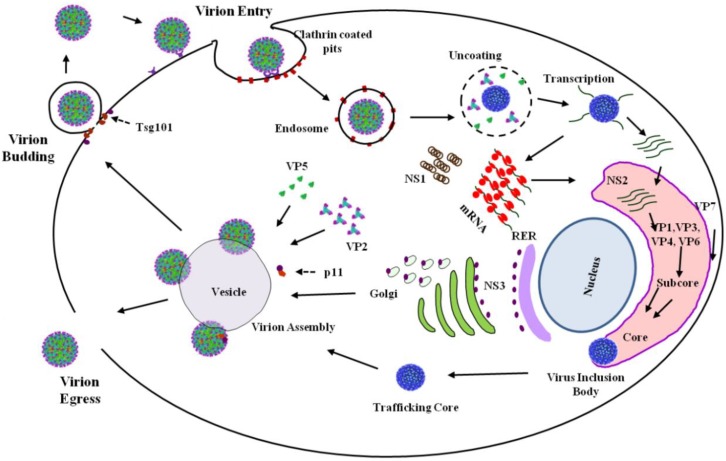
Overview of the BTV replication cycle. Virion entry is facilitated by the attachment of the outer-capsid protein VP2 to sialic acid prior to clathrin mediated endocytosis. Virion internalization leads to trafficking to endosomes where acidic pH facilitates VP5 mediated membrane permeabilization. This results in virion un-coating that releases the core into the cytoplasm. The cores become transcriptionally active, synthesizing and extruding viral mRNA for translation, leading to cellular morphogenesis via the non-structural proteins. NS1 promotes BTV ssRNA translation and subsequently forms tubules in the cytosol. NS2 assembles the viral inclusion bodies (VIBs) that concentrate viral proteins and newly synthesized ssRNAs for core assembly. Following core assembly and egress from VIBs, cores are trafficked on endocytotic vesicles by NS3 interaction with annexin/calpactin. Virion maturation is commenced by the core associating with VP5 and VP2 to form complete particles. Virions initially egress non-lytically during the early stages of infection via NS3 interacting with Tsg101, which leads to budding, prior to release by cell lysis. Adapted from [22].

BTV replication in mammals commences with the introduction of infectious virions (Figure 1) into the host blood stream during a blood-meal, infused as part of the saliva of *Culicoides* midges, during the feeding process [1]. The subsequently established primary infection occurs in mononuclear phagocytes and endothelial cells before being disseminated via the host blood stream [2]. In mammalian tissue culture cells, cell entry is mediated via adhesion to cell-surface glycoproteins [3,4] and utilizes clathrin-mediated endocytosis and pH-dependent penetration [5]. This process involves the dissociation of the outer-capsid, composed of VP2 and VP5, which then leads to the release of core in the cytosol [6]. This transcriptional activation utilizes the ten enclosed dsRNA genome segments (Figure 1), leading to the synthesis of ten single-stranded RNAs (ssRNA) that are capped and methylated [7]. Following extrusion of these transcripts from the core, they are subsequently translated into viral proteins in the cytoplasm of the infected cell. Specifically, these proteins are the seven structural proteins (VP1 through to VP7) and four non-structural proteins (NS1 through to NS4) [8,9]. NS1 has been found to be involved in virus replication and morphogenesis [10] by preferentially promoting BTV ssRNA translation to elevate viral titre [11]. NS2 is the principle component of viral inclusion bodies (VIBs) that are localized in the cytoplasm [12,13]. NS2 recruits both core proteins and newly synthesized ssRNA transcripts that are required for replication, genomic packaging and core assembly [14,15] but not the outer capsid proteins VP2 and VP5 or any other non-structural proteins [14,16]. Following assembly, cores dissociate from VIBs and subsequently mature via association with the outer capsid proteins, VP2 and VP5, prior to virion egress, a process mediated by NS3, propagating infectious virions [17,18,19] (Figure 2). NS4 has been proposed to negate the antiviral response of the host, at least in the case of serotype BTV-8 [9]. A significant advancement in our understanding of the BTV life-cycle has been the development of the first helper virus free *in vitro* T7-based reverse genetics (RG) system for BTV [20,21].

As a first step in the development of the RG system, BTV virions were purified prior to chymotrypsin treatment, which liberated the core from the outer capsid proteins (VP2 and VP5). These cores were subsequently utilized for the *in vitro* synthesis of ssRNAs. These ssRNA transcripts were then separated from the active cores prior to the former being transfected into mammalian cells. This transfection facilitated both viral protein synthesis and led to the recovery of infectious virus [20]. This system has subsequently proved malleable to the introduction of mutations into cDNA clones, using *in vitro* synthesized T7 transcripts, of either mutant or wild-type, for the elucidation of BTV biology [21]. Cumulatively, these systems allow for the elucidation and dissection of BTV assembly and maturation at a molecular level.

## 2. Highly Orchestrated Events — Capsid Disassembly and Assembly

The orchestration of capsid disassembly may be interpreted as a result of the overall structure of the BTV virion, with distinctive roles allocated and fulfilled by viral proteins contingent upon their placement within the virion architecture.

## 3. Capsid Disassembly

The two protein layers of the outer capsid possess an intrinsic instability, capable of dissociating from the core via proteolysis and mildly acidic conditions [23]. Such conditions are present following attachment and internalization of the BTV virion (Figure 2) via the clathrin-mediated endocytotic pathway and the subsequent acidification of the vesicle as it transitions to the endosome [5]. At this stage, the outer layer of VP2 may still sterically inhibit VP5 and thus regulate its activity [24]. The transition to an acidic environment may facilitate the shedding of the VP2 protein layer, having served its function of host-cell attachment and internalization, thus facilitating the exposure of the underlying VP5 protein layer. VP5 subsequently then undergoes a low-pH mediated conformational change that leads to endosomal membrane destabilization via pore formation in the confining membranes [4]. This fusogenic capacity was demonstrated in tissue culture, where recombinant protein fused to a transmembrane domain could mediate syncytia formation in *Spodoptera frugiperda* (*Sf*9) cells following low-pH shock [25]. Following membrane destabilization, it is during this stage that the dissociation of VP5 from VP7 occurs, signifying the loss of the two outer protein layers and the completion of the uncoating process, releasing the core into the cytoplasm (Figure 2). These released cores do not undergo any further disassembly, remaining intact, with the genomic RNAs contained within [5]. Consequently, the loss of the two outer protein layers and the release of the core into the cytoplasm facilitate the initiation of genome replication [26].

## 4. Viral Genome Transcription and Replication

Following release into the cytoplasm, the core, consisting of the two major proteins VP7 and VP3, the enzymatically active minor proteins VP1, VP4 and VP6, along with the 10 segments of the dsRNA genome, becomes transcriptionally active, with the 10 genomic segments never being released from the core [22]. Replication of the dsRNA genome occurs via a semi-conservative mechanism analogous to that of dsDNA replication [27]. All 10 genome segments become transcribed with the negative-sense ssRNAs of the dsRNA segments functioning as templates for the synthesis of the positive-sense ssRNA transcripts, but these are not synthesized at the same rate [28,29]. These positive-sense ssRNA transcripts are extruded into the cytoplasm as capped and methylated, but not polyadenylated, full-length mRNA copies [7,30]. Once released, these transcripts function as templates for both translation and for negative strand viral RNA synthesis to generate the genomic dsRNAs [31,32]. The 10 mRNA species facilitate the expression of 11 viral proteins, with two segments encoding two proteins each. Segment 9 encodes VP6 and NS4, which are present in different reading frames [9] and segment 10 that encodes two isoforms of NS3 (NS3 and NS3A) mediated through alternative translation start sites [33].

## 5. The Replication Complex (VP1, VP4 and VP6)

The replicase complex contained within the core is comprised of VP1, VP4 and VP6; concertedly, they are responsible for the synthesis of ssRNA transcripts from the dsRNA genome that are capped and methylated in a series of enzymatic steps. Initially, the respective enzymatic activities were assigned based upon their predicted amino acid sequence [34] and subsequently confirmed by experimental studies using *in vitro* assay systems.

VP1 is a RNA dependent RNA polymerase (RdRp) with a molecular weight of 149.5 kDa [35]. *In vitro* polymerase assays utilizing recombinant protein exhibited replicase activity that was capable of initiating BTV minus-strand synthesis *de novo*, in the absence of other viral proteins, which yielded dsRNA that was found to be RNase III sensitive, but RNase I resistant [36]. VP1 displays non‑specificity in its replicase activity, being capable of dsRNA synthesis utilizing non-viral RNA templates that had been fused with BTV sequences at the 5’ and 3’ termini, alongside Rotavirus and BTV templates [37]. However, only a minority of the potential template molecules are replicated, suggesting that alone VP1 has low replicase activity. The data indicates that the specificity of polymerase activity may also be a consequence of its association with the inner capsid of the core, acting in conjunction with the other minor proteins to associate with viral RNA to produce a conducive spatial arrangement that provides template specificity.

As previously mentioned, core derived ssRNA transcripts have been observed to be capped, in a process that occurs prior to their extrusion into the cytoplasm, and whilst VP1 facilitates the synthesis of ssRNA from the enclosed dsRNA templates, it is VP4 that has been found to be responsible for this capping modification. VP4 is a 76 kDa capping enzyme that was found capable of synthesizing cap 1 structures on ssRNA templates *in vitro* [30]. Whilst the addition of a cap to the 5’ termini of BTV transcripts allows utilization of the host-cell translational machinery, it also enhances dsRNA synthesis via VP1 when compared with ssRNA templates that lacked this feature [37]. Canonically, the prerequisite for the formation of cap structures involves three enzymatic activities of an RNA triphosphatase (RTase), guanylyltransferase (GTase) and guanine-N7-methyltransferase (N7MTase). The RTase mediates the hydrolysis of the γ-phosphate of the 5’ –triphosphate of the RNA template. Subsequently, the GTase catalyzes the formation of a 5’-5’ phosphodiester linkage between the terminal diphosphate and guanylymonophosphate. Finally the N7MTase catalyzes the addition of a methyl group to the N7 position of the terminal guanosine.

Consistent with these criteria, VP4 was shown to possess RTase [30], GTase [38] and methyltransferase activity [39]. Furthermore, in the case of Reovirus and BTV RNA transcripts, an additional nucleoside-2’-O-methyltransferase (2’OMTase) activity is required to facilitate the methylation of the 2’-hydroxyl group of the 5’ terminal nucleotide to form the cap 1 structure. Thus BTV VP4 encompasses all these essential catalytic activities for the generation of the complete 5’ terminal cap structure, and it also possesses an inorganic pyrophosphatase activity. This latter activity may facilitate VP1 polymerase processivity via the removal of inorganic pyrophosphate which may act as an inhibitor to VP1 [38].

While VP1 and VP4 activities yield positive-sense capped-ssRNAs, which serve as templates both for translation and genome replication, the dsRNA nature constraints the accessibility of the genome, which requires the unwinding of the duplex. This enables the template strands to be accessible to VP1 polymerase and facilitates its processivity as well as allowing for the dissociation of nascent ssRNA from the template strand prior to extrusion from the core. Helicases perform such functions with the intrinsic capacities for RNA binding, ATP coordination/hydrolysis and helicase function [40,41]. The last of the three minor proteins contained within the core is VP6 (36 kDa). VP6 may functions as a viral helicase. A purified recombinant VP6 has been shown capable of binding RNA duplexes with either short 5’ or 3’ overhangs or blunt-ends and unwinding these duplexes in the presence of magnesium ions and ATP [42]. VP6 contains putative functional motives, including RNA-binding and unwinding domains and ATPase activity, and when these were altered the functional activity was compromised [43]. The relevance of VP6 to BTV replication was further shown *in vivo*, where it was found to be required as part of the primary replication complex for virus recovery by the reverse genetics (RG) system [44].

Cumulatively, the three minor proteins VP1, VP4 and VP6 contained within the core cooperate and orchestrate the generation of capped and methylated ssRNA from dsRNA genomic templates, which are sequestered and shielded from the host-cell environment. 3D reconstruction of cores has shown that the intra-core organization is such that the dsRNA is associated with the minor proteins, which are associated with VP3.

## 6. Capsid Assembly and Maturation

BTV infection entails the formation of large punctuate perinuclear globules that have been termed as viral inclusion bodies (VIBs) [45], and which are viewed as sites of viral assembly [14]. VIBs are predominantly comprised of NS2 (41 kDa), which may act as a scaffold or concentrator where newly synthesized viral proteins interact with sequestered viral RNA species prior to capsid assembly and dsRNA synthesis. NS2 has been found both sufficient and necessary for VIB formation in both mammalian cell and SF9 cell expression systems, capable of forming VIB that possess a similar morphology when singly expressed as is observed during BTV infection [13,14]. Furthermore, it was found to be important for primary replication *in vivo*, as virus recovery was abolished in the absence of NS2 [46]. NS2 is the only known BTV protein that is phosphorylated *in vivo* and it is this state that may mediate VIB morphogenesis. Two serines, 249 and 259, are substrates for casein kinase 2 (CK2) [15]. This phosphorylation state regulates the propensity of NS2 to form larger aggregates, but it does not influence its capacity to bind ssRNA [15]. Whilst phosphomimetic substitutions of these serines with aspartic acid showed wild-type morphology, single or double alanine substitutions at these sites resulted in a dispersed granular morphology [15]. Thus, phosphorylation may function to regulate the interactions, and stability, of the NS2 matrix. In culture, during co-expression, NS2 is found capable of co-localizing with VP1, VP3, VP4, VP6 and VP7, although the presence of VP3 is required to facilitate VP7 recruitment [14]. In addition to its capacity for recruiting the core proteins, it can also bind newly synthesized BTV ssRNA with a higher affinity than non-BTV ssRNA [15,47,48]. These capacities and facilitations implicate VIBs as sites of core assembly and genome encapsidation.

## 7. Capsid Assembly

**Figure 3:**
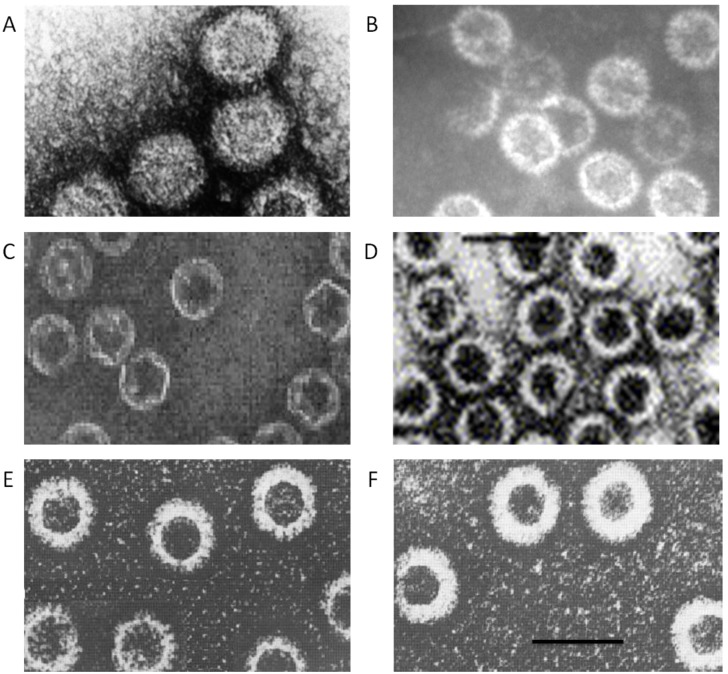
Spectrum of capsid assemblies. Transmission electron micrographs (TEM) of (**A**) authentic virions, (**B**) virus cores, (**C**) sub-core-like particles composed of VP3, (**D**) core-like particles (CLP) formed by VP3 and VP7 co-expression, (**E**) CLPs with either VP5 or VP2 added onto CLP, (**F**) virus-like particles formed by co-expression of VP3, VP7, VP5 and VP2. Bar, 100nm. Adapted from [49,54,55] and [56].

**Figure 4:**
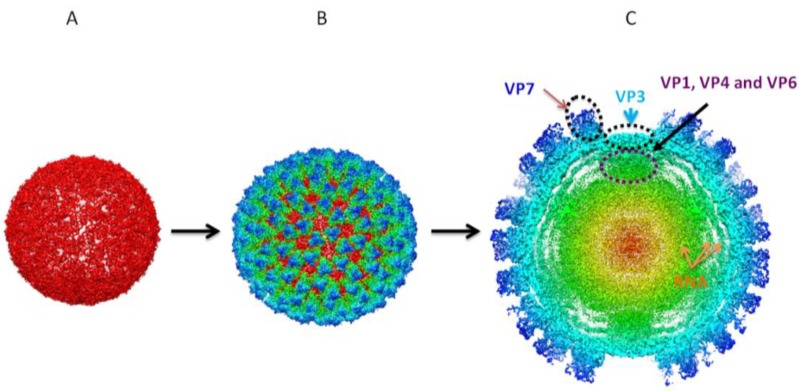
3D reconstructions of sub-core and core assemblies. 3D structural studies revealed BTV core is organized in two concentric protein layers with the sub-core composed of VP3 (**A**) forming a scaffold for VP7 to give rise to cores (**B**). (**C**) Cross-sectional representation of core assemblies shows intra-core organization of minor proteins VP1 (RdRp polymerase), VP4 (capping enzyme) and VP6 (helicase) in association with dsRNA in relation to VP3 and VP7 [57].

As previously described, *in vivo*, VIBs concentrate viral proteins and RNA in an infected host-cell, mediating, as a scaffold for, core assembly. Utilizing a recombinant protein baculovirus based expression system, it was found that when VP3 (103 kDa) was expressed on its own, or in conjunction with VP7 (39.5 kDa), it assembled into single icosahedral shells (which were of a low stability) or double-shelled icosahedral particles (with high stability) (Figure 3). 3D reconstructions of sub-cores and cores reveals that these are assembled in two concentric protein shells (Figure 4). This assembly occurred independently of the presence of the genomic RNA and minor proteins [49,50]. Given this demonstrated propensity, this system enabled, via mutagenesis, the refinement of the assembly process. The outcome of core assembly is the construction of a T = 2 sub-core composed of 60 dimers of VP3 upon which are arrayed in a T = 13 arrangement 260 trimers of VP7 that complete the core [51,52,53]. Whilst this constitutes the end-product, understanding the assembly process itself has been aided by extensive studies on recombinant core-like particles (CLPs) that have been found to possess an intrinsic propensity for autonomous self-assembly [49,54,55].

Specifically, VP3 dimers have been observed from decamers. VP3 has been denoted to possess three domains: apical, carapace and dimerization domains [52]. VP3 mutants deficient in this dimerization domain lacked the capacity for sub-core assembly, yet retained the capacity for decamer and dimer assembly [58]. This suggested an assembly hierarchy of dimers assembling to form decamers, while decamer-decamer interaction yielded sub-cores. VP7 forms trimers in the absence of VP3, yet these trimers fail to assemble into icosahedral shells. For this to occur, a scaffolding layer of VP3 is required to facilitate the deposition of VP7 trimers [59]. VP7 trimers exhibit a polymorphism in the association of VP7 subunits that give rise to five quasi-equivalent trimer species to facilitate the T = 13 arrangement of the icosahedral shell, whereby the formation of the VP7 lattice on the VP3 scaffold surface requires an exact fitting of the 260 VP7 trimers in specific order [59,60]. This is based on work with mutants that highlighted the need for correct intramolecular (within the VP7 subunit) and intermolecular (between the VP7 subunits) interactions as well as the interaction with the underlying VP3 scaffold to facilitate this assembly.

Co-expression of VP3 and VP7 yielded CLPs, reconstruction of these revealed a similar architecture and size to authentic cores (see Figure 3B), and whose assembly could facilitate the incorporation of the minor proteins VP1 and VP4 [61,62]. Whilst it was possible for CLPs to incorporate VP1 and VP4, they proved incapable of encapsidating VP6. This allows for speculation that the ssRNA synthesized during infection, for which VP6 has strong binding affinity, may be required for its incorporation, or the presence of the VIB scaffold. Interestingly, when virus derived cores are incubated with transcription buffer, conformational changes occurred, this resulted in pores on the core surface dilating, generating exit sites for the viral mRNA [63].

**Figure 5:**
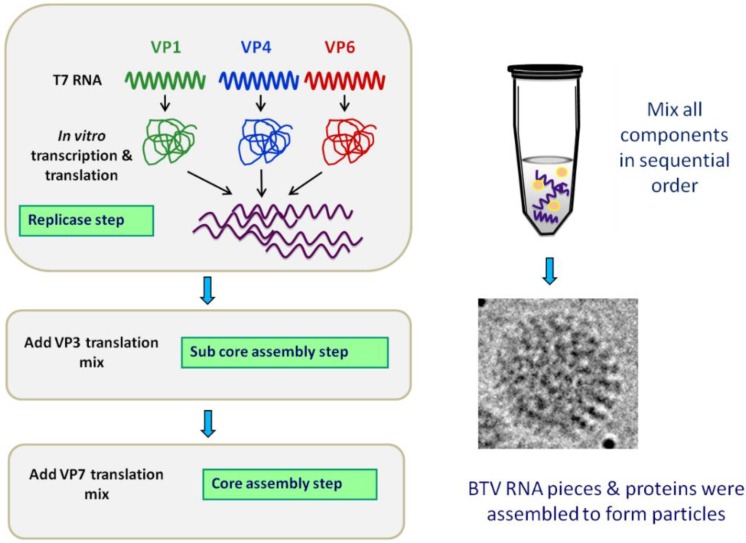
Cell-free assembly (CFA) system delineates the assembly pathway of the core particle. *In vitro* assembly of core particle involves translation of VP1, VP4 and VP6 and incubation with the ten BTV ssRNA transcripts. VP3 is added prior to the addition of VP7. The CFA system does not require any of the non-structural proteins. Adapted from [64].

These *in vivo* assembly studies have been further aided by the recent development of an *in vitro* cell free assembly (CFA) system [64] (Figure 5). Using wheat germ extract, BTV structural proteins were expressed. With the addition of the ten uncapped, positive-sense ssRNA genome segments to *in vitro* translated VP1 (RdRp), VP4 (capping enzyme) and VP6 (helicase), it was found that BTV RNA‑protein complexes could form *de novo*, and with the subsequent addition of VP3 and then VP7 that this led to the assembly of complete BTV cores, albeit with a low efficiency. In the presence of the 10 ssRNAs, generation of different viral protein combinations suggested that VP1 interacted first with VP4 and VP6, prior to its interaction with VP3, whereby VP4 would stabilize the complex. Data showed that BTV ssRNAs were essential to drive the assembly reaction. Furthermore it was found that the absence of the smallest segment, S10, resulted in a failure to incorporate the remaining segments, although protein-RNA complexes had formed, implying a role in the recruitment of the other genome segments. Following sucrose gradient fractionation it proved possible to show that these cores were indeed infectious in *Culicoides* insect vector cell culture and that the ten packaged ssRNA molecules had been converted to ten dsRNA genomic segments [64]. Furthermore, prior to the addition of VP7, the formed sub-cores of VP3 containing the minor proteins and the ten packaged ssRNA segments demonstrated sensitivity to RNase treatment, but this effect could be abrogated via the addition of VP7 [64].

These cell-free assembly and recombinant particle assembly systems have facilitated the delineation of a putative assembly order, commencing with the BTV RNA-protein complexes containing VP1, VP4 and VP6, followed by their inclusion into VP3-based sub-cores. This then subsequently recruit and serve as a scaffold for VP7 deposition, generating the stable core particles. This order of assembly events is facilitated by the viral genomic RNAs containing specific packaging signals that allow for the packaging of viral transcripts and the formation of BTV RNA-protein complexes, whilst discriminating against host cell RNAs. Studies using one RNA segment (e.g., S9) found that packaging signal was present within 276 nucleotides at the 5’ end and within 93 nucleotides at the 3’ end [44]. Given the insertions made during these experiments, a strict size limitation on segment 9 may not exist, furthermore, capping of transcripts was found not to be an essential packaging signal [44]. Once packaged into sub-cores, viral ssRNA along with the minor proteins within VIBs have been shown to be transcriptionally active. Evidence for this was provided by the observation that after the arrest of host-cell transcription via actinomycin D treatment, newly synthesized bromo-deoxyuridine labelled transcripts localized within VIBs [14].

This mode of nucleic acid encapsidation differs from the proposed mechanism employed by some phages. Phages assemble a Prohead capsid structures prior to encapsidation. Prohead I and Prohead II have similar morphologies as viewed in moderate-resolution cryo-electron microscopy (cryo-EM) reconstructions [65]. Prohead II is the structure into which the phage DNA is packaged. This differs distinctly from observations made in BTV. The current understanding derived from the CFA system is that a conformational motif formed by the interaction of the 5’ and the 3’ends of the RNA segments is necessary and sufficient for packaging. It was found that the genome assembly follows a small to large RNA segment order and that the UTRs play an important role on the regulation of packaging. Further, an RNA-RNA *in vitro* assay system indicated that there is a specific interaction between RNA segments and such interactions most likely facilitate the ssRNA incorporation into the assembling capsid [66]. In this model, the ssRNA commences association with the minor proteins, only then do these complexes allow for the commencement of encapsidation by VP3 decamers, which then amalgamate as they become incorporated into sub-core assemblies.

Interestingly within the CFA system, the presence of NS2 proved dispensable *in vitro*, this is in stark contrast to its requirement for primary replication *in vivo* [46]. This suggests that within a more hostile cellular environment *in vivo*, NS2 could concentrate viral components and protect newly synthesized ssRNA, a requirement that does not exist *in vitro* to the same extent. Furthermore, once core assembly has been completed within the scaffold of the VIBs, it is possible for these cores to dissociate and exit via channels that have been observed in VIBs by TEM [14].

## 8. Capsid Maturation

**Figure 6:**
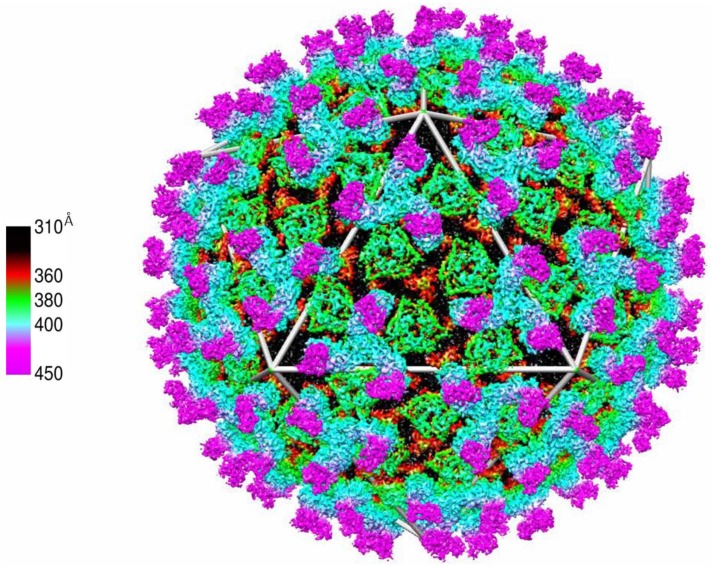
Electron-Cryomicroscopy high resolution (7-Å) image of BTV. Outer-capsid coat comprised of VP2 (cyan and magenta), the inner-capsid coat VP5 (green) and the outer-core coat protein VP7 (black and red) whereby VP3 (not visible) is occluded by the VP7 layer Adapted from [24].

While cores are assembled within VIBs, the subsequent maturation stage, which is driven by the addition of VP2 and VP5 to the cores, does not occur within VIBs [14]. The outer-capsid proteins VP2 and VP5 interact with the core via association with VP7, which comprises the outer layer of the core (Figure 6) [24,67]. 120 trimers of VP5 associate with the underlying VP7 shell but do not assume its T = 13 configuration, while 60 trimers of VP2 constitute the outermost exposed proteins [24]. Each VP2 trimer triskelion associates with four VP7 trimers on the core and globular-shaped VP5 trimers fill in the gaps created by the VP2 triskelion legs. VP5 trimers are situated above the type II and III channels of the core, which function as portals for newly synthesized mRNA transcripts to be extruded from the core. This association principle depicts protein-protein contacts of VP2 and VP5 with the underlying VP7 layer, rather than with each other. This in turn may be related to virus entry into a host cell, where each protein mediates a distinct step during the entry phase [24].

Once associated with VP2 and VP5, the transcriptionally active phase of cores may come to an end, subsequently mediating a transition to a stable dsRNA configuration, particularly when the channels utilized for mRNA extrusion are occluded by the VP5 trimers. Given the crucial nature of such a transition, it would be expected that this stage was highly regulated to abrogate the chance for pre-mature viral transcriptional shut-off. This assembly stage of maturation though, when VP5 and VP2 associated with cores, was found to have, as during core assembly itself, an intrinsic propensity for autonomous self-assembly. This was evidenced by the observation that virus-like particles (VLPs), which could mimic the morphology of authentic virions (but lacking the genomic RNA and polymerase complex), managed to assemble during the co-expression of core proteins with VP5 and VP2 in a baculovirus expression system [50,54] (see Figure 3E,F). This in turn may highlight a dependency of the virus on host-cell factors in regulating maturation via the spatial separation of VP5 and VP2 from transcriptionally active cores situated within VIBs, and facilitating maturation as the cores dissociate from VIBs and enter an egress pathway.

As VP2 and VP5 are not recruited into VIBs, experiments have shown that they instead independently interact with host-cell factors. VP5 has been shown to interact with SNARE (soluble N-ethylmaleimide-sensitive-factor attachment protein receptor) regulatory protein synaptotagmin I (Syt1) of the exocytosis pathway [68] and VP2 with vimentin, a component of intermediate filaments and cytoskeleton [16] and also with cellular exocytosis and endosomal sorting complex required for trafficking (ESCRT) pathway proteins [17]. Ultimately it may be the case that the virus utilizes its intrinsic propensity for autonomous self-assembly during the association of the core with VP5 and VP2 to recruit host factors that interact with VP5 and VP2 separately in order to facilitate egress.

In this regard, BTV maturation, where VP2 and VP5 associate with the core particle, differs from Reovirus where proteolytic processing is required. Furthermore, BTV maturation also differs from that observed in dsDNA bacteriophages or Herpes virus (HSV) maturation. Phage maturation is termed ‘expansion’ that accompanies DNA packaging, which results in a change in the dimensions of the capsid shell, increasing the internal volume without the addition of additional protein subunits [69,70]. In the case of HSV-1, capsid is formed in the infected cell nucleus as a less robust intermediate termed the procapsid. Once the DNA is packaged, the procapsid is transformed into the mature, icosahedral capsid via the angularization of the procapsid, in a process resembling Prohead expansion as in the morphogenesis of dsDNA bacteriophages [71,72,73]. To date there is no evidence that suggests capsid/core changes similar to those observed in the phages or HSV-1 occur in BTV. The core volume appears to be unaltered at low resolution cryo-EM imaging and reconstruction analysis, as the outer capsid proteins are added onto the core, or when comparing virus cores with core-like particles (CLP) that lack the genomic dsRNA segments (Figure 3, comparing B and D). More critically, the viral nucleic acids are not packaged post procapsid generation but rather the capsid (core) assembles around the viral nucleic acids, as shown in Figure 5. The culmination of these processes is the infectious BTV virion.

## 9. Virion Egress

During BTV infection both lytic and non-lytic virion release has been documented, whereby evidence exists that suggests that the host-cell environment may influence the mechanism of virion egress. Infection of mammalian cells is characterized by an extensive cytopathic effect (CPE) that predominantly, but not exclusively, leads to cell lysis, in contrast, vector insect cells demonstrate practically no CPE, concurrent to a persistent non-lytic infection [12,74,75]. Whilst non-enveloped virus release from mammalian host-cells is generally associated with cellular lysis, the significance of virion budding as a form of egress is seeing greater appreciation.

**Figure 7:**
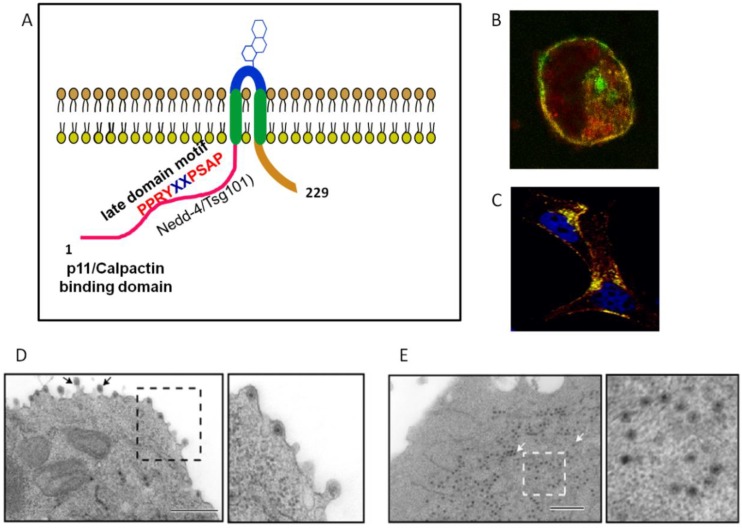
NS3 interacts with trafficking pathway protein Annexin 2 (Calpactin, p11) and ESCRT (endosomal complex required for transport) pathway protein Tsg101 and Nedd-4. (**A**) NS3 is proposed to adopt a membrane spanning topology. (**B**) Co-localization of Annexin2 (p11) and NS3 in the infected cell. (**C**) Co-localization of Tsg101 and NS3 in the infected cell. Transmission electron micrographs (TEM) of wild-type virion egress (**D**) and N-terminal NS3-p11 interacting domain mutant (**E**) that demonstrate trafficking aberration in the NS3 mutant. Adapted from [17,18,75]*.*

The role NS3 plays during virion maturation and release via the recruitment and the bridging of host factors and viral proteins is continuing to come into focus. NS3 (25.5 kDa) and its shorter form NS3A (24 kDa), which lacks the N-terminal 13 amino acids, are the only membrane-associated proteins encoded by BTV [33,76] whereby they localize to smooth-surface intracellular vesicles under TEM [77,78] (Figure 7). *In vivo*, NS3/NS3A exist in both glycosylated and non-glycosylated forms [77,79]. NS3 interacts with VP2 [17] and VP5 [68] as well as lipid raft domains [80]. It has been possible to show that co-expression of NS3 and NS3A with baculovirus-expressed VLPs in insect cells facilitated VLP release, VLPs are normally retained within the cytoplasm, and NS3 protein has also been observed at the sites of VLP release [78]. It is currently unclear whether this release is due to the cytotoxic effects as NS3 expression in mammalian or insect cells can cause when expressed alone [33], due to an ascribed function as a viroporin via the induction of membrane permeabilization [81], an attribute of the virion egress cascade.

The interaction of lipid raft domains in this context for virus maturation and egress may prove paramount. Lipid raft domains are enriched and comprised of sphingolipids and cholesterol, forming constituent parts of both the plasma membrane as well as multi-vesicular bodies (MVB) of a cell. These domains facilitate trafficking proteins, glycosylphosphatidylinositol (GPI) anchored proteins and signaling molecules to be concentrated [82,83] and may play a role in the assembly of enveloped viruses such as Ebola or HIV [84,85,86]. With regards to BTV, disruption of lipid rafts via the addition of beta-cyclodextrins, which facilitates the removal of cholesterol from cell membranes [87], did not alter BTV protein synthesis, but mediated a redistribution of VP5 and NS3 with a concurrent decrease in viral titre [68]. Furthermore, VP5 appears to also possess a capacity for associating with lipid rafts via a conserved WHXL sequence in Syt1 that a conserved WHAL motif in VP5 could bind. Alanine scan mutagenesis in WHXL resulted in a perturbation of the association of lipid rafts with VP5 [68].

The lipid phosphatidylinositol [4,5] bisphosphate (PI[4,5]P2) present within lipid rafts can interact with SNARE domains, such as found in Syt1 [88] and play a role in vesicular trafficking. PI[4,5]P2 mediates the organization of the association with the cytoskeleton and the formation of exo/endocytic cytoplasmic vesicles during vesicular trafficking [89,90]. When cellular PI[4,5]P2 was relocated to endosomal-like structures via Arf6/Q67L expression, or depletion via polyphosphoinositide 5-phosphatase IV (5ptaseIV), thus perturbing intracellular vesicle formation, BTV viral particles fail to associate with the outer surface of vesicle-like structures in the cytoplasm. Collectively, this implicates lipid raft domains as regions for virion maturation via the concerted concentration of the outer capsid proteins, possibly utilizing autonomous self-assembly during the association of the core with VP5 and VP2, prior to the facilitation of mature virion egress mediated by the role lipid raft domains play in vesicular trafficking. Such a combined particle maturation and export strategy is also utilized by other viruses, such as HIV [91,92].

Concurrent to its substantiated facilitation of maturation, the role NS3 fulfils in engaging the host cell machinery to mediate egress of BTV has also been explored. The calpactin complex, which is comprised of S100A10/p11 which forms a heterotetrameric complex with two heavy chains of Annexin A2, is involved in trafficking of proteins and membrane targeting [93]. Yeast two-hybrid analysis found that NS3 could interact with S100A10/p11, specifically, residues 1–14 of the N-terminus of NS3 [17], which are absent in NS3A. Immuno-fluorescence microscopy confirmed co-localization of Annexin2 (p11) and NS3 in the infected cell (Figure 7).

A mutant virus incapable of NS3A synthesis proved viable and competent for release from mammalian cells, whilst a mutant virus only expressing NS3A with viable genomic dsRNA synthesis, protein expression and particle assembly was severely attenuated [75]. Within the context of insect vector cell infection, both mutant viruses replicated, yet yielded a significantly lower titre. Confirmatory TEM studies determined that while wild-type virus particles were predominantly localized to within intra-cytoplasmic vesicles, mutant viruses displayed a cytoplasmically dispersed phenotype [75] (Figure 7). This study suggests, within the context of the host-background, the importance of the interaction of the N-terminus of NS3 with S100A10/p11 in mammalian cells and both NS3 and NS3A in insect vector cells for a productive BTV infection.

Furthermore, NS3 possesses two late domain motifs: PSAP and PPRY, which are also found in budding enveloped viruses such as HIV and Ebola virus [18,94]. Late domains facilitate interactions with the ESCRT pathway [95,96]. The PSAP motif of NS3 and NS3A was found capable of interacting with human tumour-susceptibility gene 101 (Tsg101) *in vitro*, as well as its homologue found in *Drosophila* [18]. Immuno-fluorescence microscopy confirmed co-localization of Tsg101 and NS3 in infected cells (Figure 7C). Mutations in the PSAP motif led to viral particles being tethered to the cytosolic membrane, apparently unable to dissociate [94]. The second late domain motif PPRY has been suggested to interact with the NEDD4-like ubiquitin ligases in HIV, although it was deemed less compatible than the native PPPY motif in Ebola virus [18]. Given NS3’s capacity to interacts with VP2 [17] and VP5 [68], this provides further evidence that NS3 fulfils the dual function of both a bridging component that allows for maturation (core association with VP5 and VP2) and also engagement with the host cell membrane trafficking machinery to facilitate virion egress [16,75].

## 10. Conclusions

Maturation is an intrinsic phase of the viral life cycle and often overlaps with egress. Our understanding of maturation has been informed by numerous studies. In this review we focused on the maturation of BTV. With the use of BTV as a surrogate for other orbiviruses, information gleaned from its study may be applicable to understanding maturation in other members of this viral genus.

In enveloped viruses, interference with glycosylation impairs maturation and egress. However, BTV lacks envelope glycoproteins, yet there appears to have been a natural evolution of a NS protein that mimics the function of some envelope glycoproteins, namely the glycoprotein NS3. BTV engages with the ESCRT machinery, both Tsg101 and NEDD4 are important (but not ALIX) as well as annexin, via NS3, and that blocking these interactions reduces virus release. EM images have shown enveloped viruses budding from the plasma membrane in a number of cells, especially during early infection, and this proportion can be changed by mutations in NS3 that prevent ESCRT engagement. Coupled with its ability to associate with lipid raft domains and the outer-capsid proteins VP2 and VP5, the capacity of NS3 to function as a bridging component, linking lipid rafts to the outer-capsid proteins that associate with the core, may be crucial. Thus, maturation in BTV appears to be very elegantly linked to its egress, maturing at sites just prior to utilizing these same sites to commence egress via the engagement of the exocytic pathways of the host cell. However, this non-lytic egress may be confined to the early stages of infection for cell to cell spread, with lytic release being the main mode of egress during the latter stages of infection. It may be hypothesized that this lytic phase is entered upon when virion production overwhelms the co-opted exocytic pathways.

Studies involving the development of techniques for understanding BTV, spanning CLPs or VLPs, *in vitro* and *in vivo* studies, have cumulatively facilitated the dissection of its life-cycle. In particular, it has aided our understanding of the molecular interactions between individual BTV components and host-factors, whereby showing how individually they influence assembly and trafficking. For example, combinations of these techniques have facilitated the discovery that NS2 is not required for *in vitro* virion assembly, yet indispensable in an *in vivo* setting. This may be due to its role as a viral concentrator within the dynamic environment of the cytoplasm. Current gaps in our understanding of how cores egress from VIB remain, yet with the extensive array of model systems available it is but a question of time before these too are filled. Thus, the essential roles the non-structural proteins play in virion maturation may offer novel targets for pharmacological interventions.

In summary, these processes have proven to be very informative and have enriched our understanding of virus maturation, providing non-parochial insights, specifically, highlighting virus ingenuity and the capacity for encoding multi-functional proteins. Techniques described here have proven transferable and will continue to facilitate the exploration of diverse viruses for the development of possible intervention strategies.
